# Retraction: Sustained Exposure to the Widely Used Herbicide Atrazine: Altered Function and Loss of Neurons in Brain Monoamine Systems

**DOI:** 10.1289/ehp.7783RET

**Published:** 2012-07-02

**Retraction of: Environ Health Perspect 113:708-715 (2005); doi:10.1289/ehp.7783**

The Office of Research Integrity has determined that Mona Thiruchelvam falsified and fabricated cell count data presented in “Sustained Exposure to the Widely Used Herbicide Atrazine: Altered Function and Loss of Neurons in Brain Monoamine Systems” [Rodriguez VM, Thiruchelvam M, Cory-Slechta DA. Environ Health Perspect 113:708-715 (2005)]. The Editor-in-Chief has retracted the paper.

For additional information, please go to: http://www.gpo.gov/fdsys/pkg/FR-2012-06-28/html/2012-15887.htm

